# Impact of administered amount of peptide on tumor dosimetry at the first cycle of peptide receptor radionuclide therapy (PRRT) in relation to total tumor somatostatin receptor expression

**DOI:** 10.1186/s13550-023-00997-0

**Published:** 2023-05-19

**Authors:** Ulrika Jahn, Ulrike Garske-Román, Mattias Sandström, Mark Lubberink, Anders Sundin

**Affiliations:** 1 Department of Surgical Sciences, Nuclear Medicine and PET, Uppsala University Hospital, 751 85 Uppsala, Sweden; 2 Department of Blood and Tumor Diseases, Uppsala University Hospital, Uppsala, Sweden; 3 Department of Medical Physics, Uppsala University Hospital, Uppsala, Sweden; 4 Medical Imaging Centre, Uppsala University Hospital, Uppsala, Sweden; 5 Department of Immunology, Genetics and Pathology, Uppsala University, Uppsala, Sweden

**Keywords:** Peptide, SUV, Small intestinal NET, Pancreatic NET, Somatostatin receptor, PRRT

## Abstract

**Background:**

The accumulation of ^177^Lu-DOTATATE might be influenced by the amount of administered peptide in relation to the tumor somatostatin receptor expression. The effect of the administered peptide mass on the resulting absorbed dose in tumors and normal organs has not previously been assessed in relation to the patients’ tumor load.

**Method:**

Patients with small intestinal (*n* = 141) and pancreatic (*n* = 62) neuroendocrine tumors (NETs) who underwent PRRT were selected for retrospective evaluation. All patients had received 7.4 GBq ^177^Lu-DOTATATE, and the amount of administered peptide in the preparation varied from 93 to 456 µg. The absorbed dose in tumors and normal tissue at the first PRRT cycle was calculated, based on SPECT-measurements at day 1, 4, and 7 post-infusion. The total tumor somatostatin receptor expression (tTSSTRE) was calculated on SPECT after 24 h by multiplying the functional tumor volume, delineated by 42% cut-off VOIs of the highest activity, with the SUVmean for the respective tumor VOIs. Spearman’s rank correlation analyzed any relationship between the administered amount of peptide and the absorbed dose in tumors and normal organs, in relation to the patients’ tTSSTRE.

**Results:**

There was no correlation between the amount of peptide and any of the tested parameters in relation to tTSSTRE.

**Conclusion:**

In this retrospective analysis, no correlation between the amount of administered peptide in the ^177^Lu-DOTATATE preparation and the absorbed radiation doses in tumors and normal tissues was demonstrated in relation to the total tumor SSTR expression.

## Introduction

Peptide receptor radionuclide therapy (PRRT) has become a well-accepted second- and third-line treatment for patients suffering from locally advanced and disseminated, well-differentiated somatostatin receptor (SSTR) positive neuroendocrine tumors (NETs) [1–6], whereby a radiolabeled somatostatin analog (SSA) is administered as treatment cycles every 8–12 weeks. Extensive work in the late twentieth century focused on developing the most effective SSA together with the best chelate for the chosen radiolabel (^90^Y or ^177^Lu) [7–9] in order to attain the most effective tumor cell internalization of the peptide-receptor complex [10–12]. ^68^Ga-labeled DOTATOC, DOTATATE and DOTANOC are used for PET/CT imaging of NETs in the clinical routine. To date ^177^Lu-DOTATATE is the most frequently used preparation for PRRT [6]. Radiolabeled DOTATATE mainly interacts with the somatostatin receptor 2 (SSTR_2_), but partially also with the somatostatin receptor 5 (SSTR_5_) [8, 13]. Soon after internalization, the ligand is rapidly dissociated from the receptor, which is recycled or destroyed*. *In vitro studies have shown that about half of the dissociated radioligand remains intracellularly, while the rest is recirculated or metabolized [10, 12]. The PRRT preparation is regularly administered as an intravenous infusion, although administration through the liver artery also has been tested [14]. While the PRRT preparations mainly accumulate in the tumors due to their SSTR abundance, the absorbed doses to the most radiosensitive normal tissues, mainly bone marrow and kidneys, need to be monitored [15, 16], as also required by the regulatory authorities.

For patients with tumor spread beyond surgical intervention, treatment with a systemic slow-release SSA preparation comprise the first line therapy in low-grade small-intestinal NETs (SI-NETs) and pancreatic NETs (P-NETs). It is known that continuous treatment with SSA upregulates the receptors in both tumors and normal tissues, although to a larger extent in the tumors [17]. For patients receiving treatment with long-acting SSAs, both the European Neuroendocrine Tumor Society (ENETS) and the European Association of Nuclear Medicine (EANM) recommend that both diagnostic (^68^Ga-DOTA-SSA-PET/CT) and therapeutic (PRRT) procedures with radiolabeled SSAs should be performed shortly before the patient’s next treatment with long-acting SSAs to minimize a competitive blocking of the tumor SSTRs. This advice is based on the assumption of a competitive binding between labeled and unlabeled SSA at the SSTR site that would decrease the effect of PRRT [18, 19]. However, this routine is questioned by Bozkurt et al. in the EANM guidelines for PET/CT with ^68^Ga-DOTA-SSA and ^18^F-DOPA [20, 21]. Already in 1993, Dörr et al., questioned the recommendation, based on their finding that the tumor detection was improved by i.v. administration of a short acting SSA to five patients before SSTR scintigraphy with indium-111 pentetreotide [22]. In a later study, short-acting octreotide was injected intravenously immediately before ^68^Ga-DOTATOC-PET/CT and showed a dose-dependent decrease in the normal tissue uptake. In tumors, however, there was an increased uptake at a low dose (50 µg), but not at a high dose (500 µg), except in one patient with a very large P-NET [23]. The results of these reports did, however, not impact the recommendations by the ENETS, nor those by the EANM. The objective of the present study was to assess the potential impact of the amount of administered peptide in the ^177^Lu-DOTATATE preparation on the absorbed radiation dose in tumors and normal tissues in relation to the patients’ total tumor SSTR expression (tTSSTRE) measured at the first PRRT cycle.

## Material and methods

### Patients and PRRT protocol

Between 2006 and 2009, PRRT was administered according to a compassionate-access program (Swedish Medical Products Agency), and from 2009, the patients were included in the prospective ^177^Lu-DOTATATE trial (EudraCT 2009-012260-14), with prolongation until the commercial ^177^Lu-DOTATATE-preparation (Lutathera®) was introduced (September 2018). The eligibility for the patient to receive PRRT was based on the findings of sufficient tumor SSTR expression, higher than that in the normal liver, based on SSTR-scintigraphy (OctreoScan™). Data were extracted from those SI-NET and P-NET patients who received 7,4 GBq ^177^Lu-DOTATATE at their first PRRT cycle and for whom the administered amount of peptide (µg) and absorbed dose to tumor (Gy) were retrievable.

**Fig. 1:**
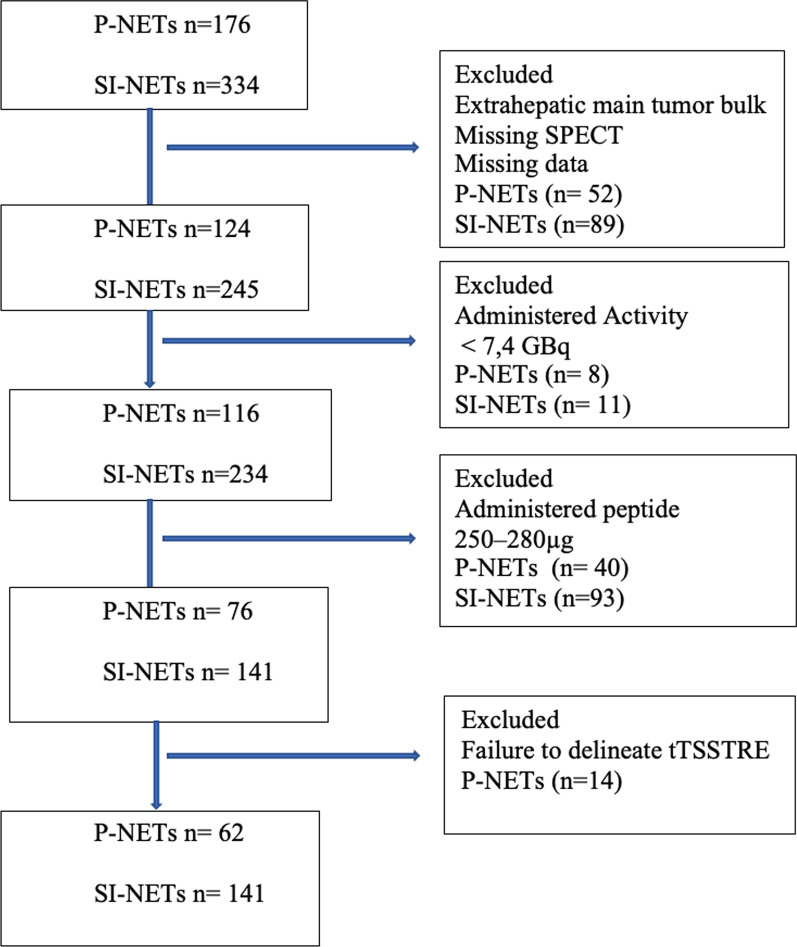
Diagram outlining the selection of patients with SI-and P-NET for the final analyses

Only patients with abdominal metastases (mainly liver metastases) were selected in order to include the vast majority of tumors within the field-of-view of the abdominal SPECT examination during PRRT dosimetry. Consequently, mainly liver metastases were evaluated, but also primary tumors and abdominal and retroperitoneal lymph node metastases within the field-of-view. At Uppsala University Hospital, 510 patients with SI-NETs and P-NETs were treated with PRRT between April 2006 and September 2018 (Fig. 1). One hundred forty-one patients were excluded because of missing SPECT examinations in the image archives, missing information about which SPECT/CT scanner was used, a lack of liver metastases (main tumor bulk outside the field of-view of SPECT), PRRT inclusion based on PET instead of SSTR-scintigraphy (OctreoScan™), and missing data on body weight. Nineteen patients who received less than 7.4 GBq were similarly excluded.

The peptide was a kind gift from Prof. Eric Krenning. Lutetium-177 was purchased from IDB, Holland BV, and labeling was performed in-house.

Each batch of ^177^Lu was used for labeling of DOTATATE and administration of PRRT either on the day of arrival or 4 days later, resulting in high and low specific activity for the first and second ^177^Lu-DOTATATE-preparation, respectively. Consequently, when using the first ^177^Lu-DOTATATE-preparation of the week, the patients received amounts of peptide in a lower range (high specific activity) compared to those receiving PRRT with the second preparation of the week, with a peptide mass in a higher range (low specific activity). In this exploratory analysis, as an attempt to further increase and contrast the differences between the administered amount of peptide in the “low-peptide” and “high-peptide” groups, patients receiving peptide amounts in the 250–280 µg interval were excluded (*n* = 133). Thus, the patients receiving either 93–249 µg or 281–456 µg of peptide remained. The estimation of the patients’ total tumor somatostatin receptor expression (tTSSTRE) on the 24-h SPECT examination (see below) failed in fourteen P-NET-patients because of visual discrepancies between the software-based delineation of the functional volume versus the morphological tumor volume on CT. Consequently, a total of 203 (40%) SI-NET and P-NET patients (*n* = 141 and 62 respectively) were included for analysis. (Fig. 1).

### Peptide receptor radionuclide therapy (PRRT)

PRRT was administered according to previously published procedures by applying a dosimetry tailored treatment protocol. As many cycles as possible were administered until 23 Gy absorbed dose to the kidneys or 2 Gy to the bone marrow [4, 24, 25] was reached. PRRT was administered as 7.4 GBq of ^177^Lu-DOTATATE in 100 mL of saline that was infused intravenously for 30 min parallel with an ongoing intravenous infusion (2 h) of mixed amino acid solution for kidney protection starting before ^177^Lu-DOTATATE-administration. ^177^Lu-DOTATATE was administered according to the ENETS recommendations, observing a 4–6-week interval after treatment with long-acting SSAs and before start of PRRT [19].

### Administered amount of peptide

From the patients’ records, the amount of peptide administered to each patient in the ^177^Lu-preparation was retrieved. This varied in the P-NET group between 170–373 µg and in the SI-NET group between 93–456 µg.

### Twenty-four-hour SPECT examination

Because the retrospective data were collected from PRRT performed over a decade, SPECT imaging was done by four types of gamma cameras, Millenium VG, Infinia, Discovery 670 and Discovery 870 CZT (all GE Healthcare), all equipped with a CT scanner used for attenuation correction. Imaging on the VG scanner utilized 60 angles with 60 s for each frame, and for the other three systems 120 angles with 30 s for each frame was used. The energy window for the CZT scanner was 208 keV (± 6%), for the VG scanner the 113 keV (± 10%) and 208 keV (± 10%) windows were summed, while for the Infinia and Discovery 670 cameras, a 208 keV (± 10%) window was used. The collimators were MEHR for the CZT scanner and MEGP for the three other systems.

### Total tumor somatostatin receptor expression (tTSSTRE)

The patients’ total tumor somatostatin receptor expression (tTSSTRE) was assessed from the 24-h SPECT examination using a non-commercial research version of Affinity Viewer 3.0 (HERMES Medical Solutions AB., Stockholm, Sweden). SPECT images were converted to SUV using a converting calibration procedure. Semiautomated VOIs were then generated to outline all tumors within the field-of-view of the SPECT examination. For tumor conglomerates, a software “splitter” tool allowed for larger VOIs to be split into smaller VOIs, representing single tumors and/or homogenous tumor areas. In the next step, a 42% cut-off of the highest SUV in each VOI was applied, and the resulting functional volume (FV) of each VOI was registered, together with their respective SUV. For each VOI, the SUVmean was multiplied by the FV to achieve its tumor somatostatin receptor expression (TSSTRE). In the final stage, the TSSTRE for all tumor VOIs were added to form the patient’s total tumor somatostatin receptor expression (tTSSTRE). The intention was to apply an identical SUV cut-off value for the tumor VOI measurements in all patients. However, when performing a pilot test on P-NETs (*n* = 48) and SI-NETs (*n* = 73), it was found necessary to adapt separate SUV cut-off values for SI-NET and P-NET patients. In the subsequent analysis of the study cohort, it became obvious that within the groups, patients with small and large tumor load required different SUV cut-off values. Thus, separate SUV cut-off values were applied for patients with high tumor load (SI-NETs SUV 5.5 and P-NETs SUV 7.1) but the same for all patients with low tumor burden (SUV 3.5). As described above, the predefined cut-off settings failed to accurately delineate the tumors in 14 P-NET patients. In the final analyses, the tTSSTRE was divided into three groups; low (< 5000 (SUV x mL), *n* = 106), median (5000–15,000 (SUV x mL), *n* = 56) and high (> 15,000 (SUV x mL), *n* = 41).

### Dosimetry

Dosimetry for normal organs and tumors at the first PRRT cycle was based on SPECT/CT acquisitions at 24, 96 and 168 h after ^177^Lu-DOTATATE administration, and was performed according to earlier published procedures [4]. Tumor dosimetry for each patient was calculated for the two to three tumors with the highest uptake, and the median absorbed tumor dose was applied in the further statistical analysis.

### Statistics

Spearman’s rank correlation was applied to analyze correlation between all tested parameters: amount of administered peptide, tTSSTRE, median absorbed dose in tumors, kidneys, spleen, liver and absorbed dose ratios (tumor-to-kidney ratio, tumor-to-spleen ratio, and tumor-to-liver ratio). tTSSTRE was calculated in regards to three categories, low, medium and high and the absorbed dose versus amount of peptide of all parameters was tested within each category using regression analysis.

## Results

**Fig. 2:**
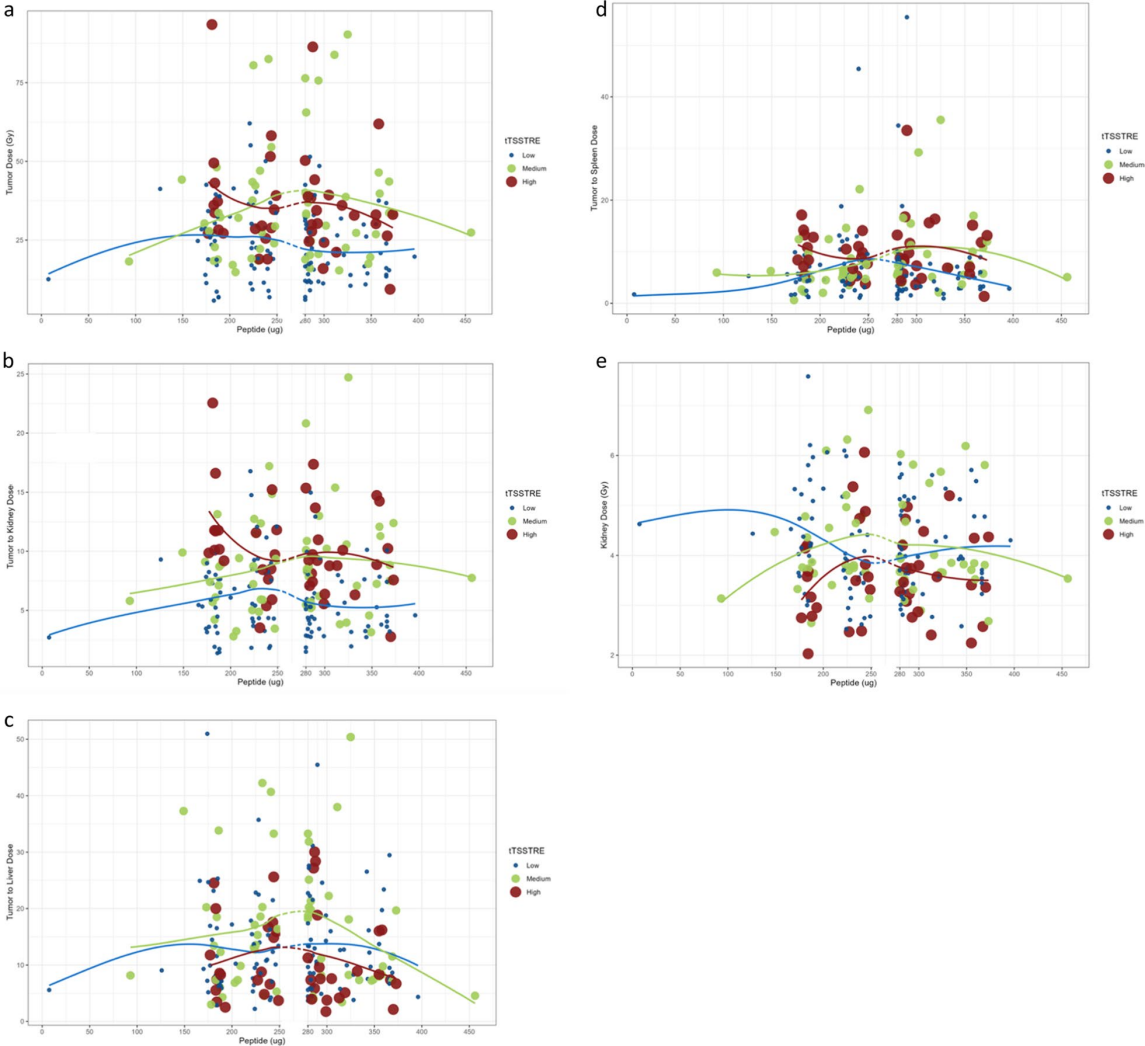
Blue dots represent the patients in the lowest tTSSTRE group (values < 5000 (SUV x mL), *n* = 106). Green dots represent the patients in the medium tTSSTRE group (values 5000–15,000 (SUV x mL), *n* = 56). Brown dots represent the patients in the largest tTSSTRE group (values > 15,000 (SUV x mL), *n* = 41). The blue, green and red lines represent the fitted line for the peptide values of each tTSSTRE group. **a** Regression analyses whereby the mean tumor absorbed dose is plotted against the administered amount of peptide at the first PRRT cycle in respect to the patient`s total tumor somatostatin receptor expression (tTSSTRE). **b** Correlation between amount of administered peptide and the tumor-to-kidney absorbed dose ratio, with respect to the total tumor somatostatin receptor expression (tTSSTRE). **c** Correlation between amount of administered peptide (µg) and the tumor-to-liver absorbed dose ratio, with respect to the total tumor somatostatin receptor expression (tTSSTRE). **d** Correlation between amount of administered peptide (µg) and the tumor-to-spleen absorbed dose ratio, with respect to the total tumor somatostatin receptor expression (tTSSTRE). **e** Correlation between amount of administered peptide and the mean kidney absorbed dose with respect to the total tumor somatostatin receptor expression (tTSSTRE)

In the Spearman’s Rank correlation, no impact of the administered amount of peptide was found on any of the analyzed parameters: median absorbed dose in tumors, median absorbed dose in kidneys, spleen and liver and absorbed dose ratios (tumor-to-kidney, tumor-to-spleen, and tumor-to-liver) in relation to tTSSTRE (data not shown). Neither did the administered amount of peptide impact the same parameters in the further regression analysis of each group of tTSSTRE (low, median and high) (Fig. 2a–e).

## Discussion

This study investigated the possible impact of the amount of peptide, administered in the ^177^Lu-DOTATATE preparation, on the absorbed dose in tumors and normal tissues at the first PRRT cycle, and in relation to the patients’ total tumor SSTR expression (tTSSTRE). The patients who underwent PRRT on the day of ^177^Lu delivery received a ^177^Lu-DOTATATE-preparation with an amount of peptide in the lower range, whereas patients who underwent PRRT 4 days after ^177^Lu delivery received a preparation with an amount of peptide in the higher range. The patients’ tumor load was assessed on the SPECT-examination performed 24 h after ^177^Lu-DOTATATE-infusion. In order to assure that almost all tumors were included in the field-of-view of the abdominal SPECT, patients with mainly liver metastases were selected. In this retrospective setting, the 24 h SPECT examination was utilized for the calculation of the tTSSTRE, although at this time point, it is uncertain what the remaining tumor radioactivity might represent, regarding the process of ligand-receptor dissociation and recycling and the metabolism of the ^177^Lu-DOTATATE molecule [12, 26–28]. Thus, in a prospective study, the optimal time point to calculate the tTSSTRE would be approximately 3 h after ^177^Lu-DOTATATE-infusion when the tumor uptake is the highest [29].

In this study, it was not possible to demonstrate any influence from the administered amount of peptide in the ^177^Lu-DOTATATE-preparation on the absorbed dose in tumors or in the normal tissues (kidney, spleen, liver), or on the corresponding tumor-to-normal tissue ratios. No relation was found between the administered amount of peptide and the patients’ total tumor somatostatin receptor expression (tTSSTRE) either. Our findings are in contrast to two recent reports, one comparing standard peptide amounts versus both high and low amounts of peptide administered in the ^177^Lu-DOTA-3-iodo-Tyr3-octreotate (^177^Lu-HA-DOTATATE) preparation [30] and another comparing radioactive uptake of ^177^Lu-HA-DOTATATE with or without postponing the long-term SSA medication [31]. Siebinga et al. studied 13 patients receiving 15 cycles with a high peptide amount of 346 ± 33 μg (mean ± SD) and found decreased uptake in tumors, spleen and kidney as compared to 15 cycles administered with a standard peptide amount of 178 ± 8.8 μg, and with similar uptake in liver, blood and bone marrow. In 15 patients receiving 15 cycles with low peptide amount of 109 ± 6.6 μg, decreased uptake was found in tumors and increased uptake was found in spleen, as compared to 15 cycles administered with a standard peptide amount of 202 ± 15 μg, and with similar uptake in kidney, liver, blood and bone marrow [30]. In line with their findings, Veerman et al. reported a clear decline in liver and spleen uptake of ^177^Lu-HA-DOTATATE in patients continuing long-acting SSAs during PRRT, as compared to those who stopped SSAs before treatment start, although the uptake in tumors, kidneys, bone marrow and blood pool was similar between groups. These conflicting results are likely explained by the different methodology regarding the choice of PRRT cycles, peptide amounts, effect metrics (uptake versed dosimetry), NET types and number of patients. While Siebinga et al. retrospectively compared different PRRT cycles in the same patients, our study exclusively focuses on the first PRRT cycle in order not to risk confounding factor of therapy effects on the dose response. In contrast to the present study, which compared the absorbed doses in the tumors and normal tissues based on 7-day dosimetry, both Siebinga et al. and Veerman et al. reported differences in tissue uptake on the 24 h SPECT/CT. Neither did Siebinga et al. or Veerman et al. relate their results to the patient’s total SSTR expression, as in the present study.

Our findings contrast those in earlier imaging studies, such as SPECT with ^111^In-pentreotide [22] and ^68^Ga-DOTATOC PET/CT [23] where generally lower amounts of injected peptide are used compared to PRRT, which is a probable confounder in the comparison.

Further, except for the differences in the administered amounts of peptide between imaging studies and PRRT reports (also including the present one), the time point for measurements is diverse. Thus, PET/CT imaging at one-hour post-injection of ^68^Ga-DOTATOC/TATE differs very much from the SPECT/CT registrations starting at 24 h after initiation of PRRT. Considering the time frame for the receptors to resurface (7 to 24 h) [12, 27, 28], ^68^Ga-DOTATOC/TATE-PET/CT mainly registers the influx of the ligand-receptor complex before any dissociation or metabolization has occurred. SPECT/CT performed after 24 h will, by contrast, encounter processes of receptor ligand dissociation completed within 6 h, and both ligands and receptors recirculate [12]. It is further uncertain how much of the radioactivity that is left in the tumor cells 24 h post injection, as it was shown by Anderson et al. that only 50% of the initially incorporated radioactivity remains after 12 h when using carcinoid cells cultures in vitro [10]. Consequently, the radioactivity registered on SPECT at 24 h most likely represented a fraction of the initial radioactivity internalized with the receptors. This will consequently affect the assessment of tTSSTRE, based on 24-h SPECT/CT, as compared to a similar estimation using ^68^Ga-DOTA-SSA-PET/CT at 1 h, and also impact the measurements of tissue uptake at 24 h SPECT [30] versus that of absorbed dose based on subsequent SPECT 1 to 7 days as in the present study. Additional factors adding to the differences between the PET-experiments and the therapy setting are the intravenous amino acid infusion during PRRT for kidney protection that may have impacted the ^177^Lu-DOTATATE biodistribution at PRRT versus that of ^68^Ga-DOTATOC/TATE at PET/CT, and the difference between the two preparations regarding the radiometal and the peptide, since any change of the radioligand alters its affinity and behavior [8].

Dosimetry during PRRT was performed according to procedures developed at our center and have been shown to be reliable for normal organ dosimetry [4]. Up to three of the largest tumors with the highest 177Lu-DOTATATE uptake on SPECT per patient were included for dosimetry. The precision in these tumor measurements, performed with a technique primarily developed for normal tissues, with homogenous distribution, may not have been as high as desirable for the present assessment. Although homogeneous tumor areas were chosen for tTSSTR analysis, minor irregularities in the tumor uptake may have influenced the study results. Further, the influence of the partial volume effects is yet a factor of concern. Probably, this predominately affected the group of patients for whom low cut-off, rather than high cut-off SUV VOIs, were applied in the SPECT examinations for tumor delineation. Visually, it was however clear that many small tumors were excluded from the tumor VOIs when the SUV cut-off step was applied. There were wide variations in tumor load and SSTR expression between our P-NET and SI-NET patients, and also within each tumor group. Thus, in order for the semiautomated soft-ware delineation (tumor VOIs) to correspond to the morphological tumor burden on CT, the tTSSTRE calculations required the use of two different SUV cut-off thresholds, in P-NET and SI-NET-patients, with large tumor load, but similar cut-off was feasible for all patients with a low tumor burden. This was not unexpected, considering our previous findings of different absorbed doses to tumor in P-NETs and SI-NETs [32]. Thus, the fact that it was not possible to apply one SUV cut-off threshold for all patients, may accordingly have introduced a bias in the tTSSTRE data.

To achieve the tTSSTR, we applied 42% iso-contour VOIs, originally adapted to delineate tumors on FDG-PET to accomplish a VOI size that fits the tumor size on CT. There is thus no support that the 42% iso-contour tumor VOIs are optimal in the present setting of ^177^Lu-DOTATATE-SPECT and other iso-contour percentages may be more appropriate. In the present evaluation, the tumor VOIs on SPECT fairly well-corresponded to tumor size on CT, and 42% iso-contour VOIs were therefore applied as a starting point for our assessment.

To our knowledge, this is the first study to examine the potential influence of the amount of administered peptide in the ^177^Lu-DOTATATE preparation, on the absorbed dose in the tumors, and also taking the patient’s tTSSTRE into account. Limitations of this study, including 40% (203/510) of our P-NET and SI-NET patients, are its retrospective design over a decade with use of different SPECT/CT gamma cameras, the non-standardized SUV cut-off applied for tumor delineation on the SPECT/CT examinations, assessment of tTSSTRE at 24 h rather than during the tumor uptake peak at 3–4 h, and that the dosimetry technique developed for normal organ dosimetry was applied to also calculate the absorbed dose in the tumors [29].

In conclusion, the amount of administered peptide in the ^177^Lu-DOTATATE preparation did not correlate to the absorbed dose in the tumors and normal organs and was unrelated to the patients’ total tumor somatostatin receptor expression (tTSSTRE). Given the sparse evidence in the literature of the impact of the administered peptide mass at PRRT in well-differentiated NETs, our findings warrant further investigation.

## Data Availability

The dataset used and/or analyzed during the current study are available from the corresponding author on reasonable request.

## Abbreviations

PRRT: Peptide receptor radionuclide therapy
SPECT: Single-photon emission computed tomography
CT: Computed tomography
SUV: Standardized uptake value
VOI: Volume of interest
FV: Functional volume
NET: Neuroendocrine tumor
SI-NET: Small-intestinal NET
P-NET: Pancreatic NET
PET/CT: Positron emission tomography /computed tomography
SSA: Somatostatin analog
SSTR: Somatostatin receptor
tTSSTRE: Total tumor somatostatin receptor expression
ENETS: European Neuroendocrine Tumor Society
EANM: European Association of Nuclear Medicine
